# MISAPSY: childhood maltreatment, food insecurity, psychological distress and professional integration among socioeconomically disadvantaged young adults – a mixed-methods study protocol

**DOI:** 10.1186/s40359-026-04292-0

**Published:** 2026-03-13

**Authors:** Maud Cappelletti, Joris Mathieu, Maeva Musso, Gérard Shadili, Isée Bernateau, Ariane Bazan, Marion Robin, Aziz Essadek

**Affiliations:** 1 Université de Lorraine, INTERPSY, Nancy, F-54000 France; 2 Laboratoire CRFDP UR7475, Nutrition Department, Rouen University Hospital Center, Rouen, 76000 France; 3 Hôpitaux Paris Est Val de Marne, Saint-Maurice, France; 4 Department of Adolescence and Young Adult Psychiatry, Institut Montsouris, Paris, 75014 France; 5 Laboratoire CRPMS UR3522, Université Paris Cité, Batiment Olympe de Gouges – 8 rue Albert Einstein, Paris, 75013 France; 6 Institut Universitaire de France, Paris, France

**Keywords:** Food insecurity, Trauma, Adverse childhood experiences, Mental health, Young adults, Mission locale

## Abstract

**Background:**

The MISAPSY study (Childhood Maltreatment, Food Insecurity, Psychological Distress and professional integration among socioeconomically disadvantaged young adults) aims to model the interrelations between child maltreatment, psychological distress and food insecurity, among precarious young adults enrolled in French integration services (Mission Locale). This population is exposed to multiple forms of social and material precarity. While food insecurity constitutes a tangible and increasingly prevalent dimension of precarity, it remains underexplored. By examining food insecurity alongside traumatic experiences and psychological distress, the study seeks to develop transferable explanatory models to inform the design of targeted prevention and support programs for young people in vulnerable situations.

**Methods/design:**

A triaxial methodology approach is implemented: (1) a cross-sectional study using standardized questionnaires to identify associated factors; (2) a qualitative study based on semi-structured interviews exploring psychologists’ subjective experiences of supporting these young people; and (3) a longitudinal comparative interventional study evaluates two different support programs (food aid only and combination of food aid and psychological support) for young adults enrolled in an insertion pathway. Outcomes include changes in psychological well-being and in food insecurity. Quantitative analyses will include descriptive and multivariate statistical tests using R, while qualitative data will be analyzed through thematic analysis with NVivo. Intervention effects will be examined using pre/post comparisons, mixed models, and appropriate statistical corrections.

**Discussion:**

This study is expected to highlight the high prevalence and interrelated nature of food insecurity, psychological distress, and traumatic experiences in this population. It is hypothesized that the combined intervention will show greater benefits for mental health status. MISAPSY aims to contribute to integrated, trauma-informed and food-security-oriented models to guide practitioners and policymakers in addressing the specific needs of socioeconomically disadvantaged young adults.

**Trial registration:**

This study is registered with the Biomedical Research Identification Number (n°ID-RCB) assigned in France by the National Agency for the Safety of Medicines and Health Products (ANSM): 2025-A01928-41, and have received the approval from the Committee for the Protection of Persons Ile-de-France XI and two Ethics Committees. This study was also registered on ClinicalTrials.gov on February 20, 2026 (NCT07427524). The full protocol and prespecified outcomes are publicly accessible through this registry.

**Supplementary Information:**

The online version contains supplementary material available at 10.1186/s40359-026-04292-0.

## Background

The Mission Locale (ML) is a French public institution designed to promote the professional and social integration of young people who encounter difficulties in the areas of employment, training, health or housing. Eligibility criteria for ML enrollment are: being aged 16–25 years; no longer being a student; encountering at least one difficulty related to the areas mentioned above, regardless of immigration status [1]. Beyond their primary objective of removing barriers to employment, these structures promote autonomy and independence. From a multidisciplinary professional perspective, MLs offer various programs related to education, training, schooling, health, social services and judicial prevention, within a holistic approach to the individual [2]. The trajectories of young adults supported by ML services are diverse but vulnerability factors are frequently observed, including school dropout, material insecurity, lack of employment or financial resources, multiple difficult life events in childhood and social isolation [3].

Young adulthood constitutes a transitional period marked by complex psychological adjustments, which can be hindered by adverse social contexts or significant crises. The global COVID-19 pandemic had a resounding and multifactorial impact worldwide [4] and led to an unprecedented increase in precariousness. In France, about 17% of 18–29-year-olds live below the poverty line and nearly half of poor people are under 30 years old [5, 6]. Youth in precarious situations frequently experience multiple psychosocial risk factors that affect their mental health. The deterioration of mental health among these vulnerable young people is a major concern, especially because the levels of depression and anxiety assessed in this population are notably high and continue to worsen [7, 8].

Food Insecurity (FI) can be defined as uncertainty about regular access to a sufficient quantity of food, of adequate quality to ensure a healthy and fulfilling life [9]. It is a tangible dimension of material precariousness. This increasing form of instability acts as a chronic stressor, due to an inability to meet basic needs [10, 11]. It has detrimental effects on psychological and emotional functioning. Regardless of levels of economic development, an analysis of data from 149 countries reports an increased prevalence of mental health disorders in contexts of moderate to severe FI [12]. Furthermore, a recent meta-analysis highlighted a significant association between FI and symptoms of depression, anxiety, perceived stress and sleep disorders [13]. Thus, FI appears to be one of the main social determinants of mental health, especially among 18–35-year-olds, showing strong associations with anxiety and depressive disorders and suicide risk, as highlighted by several studies [14–16]. In addition, FI has been identified as a predictor of child maltreatment within households [17].

The associations between low levels of food security and childhood maltreatment are bidirectional. Child abuse, which can take many forms, constitutes major Adverse Childhood Experiences (ACEs). The consequences of an accumulation of ACEs are numerous, including physical and mental health disorders, and can lead to premature death [18, 19]. Exposure to ACEs also increases the risk of experiencing FI in adolescence and adulthood. Certain forms of ACEs, such as emotional neglect, further exacerbate this risk, regardless of socioeconomic status [20, 21]. Childhood maltreatment, through its interpersonal nature, constitutes a potential precursor to the development of trauma, particularly in its complex form, which is linked to possible persistent alterations in the affective, relational and self-image spheres. It is also an identified risk factor for the emergence of other mental disorders that persist into adulthood [22–24]. In this research, we consider 5 types of ACEs: emotional abuse, physical abuse, sexual abuse, emotional neglect, physical neglect.

Thus, instability in food access cannot be understood solely as a social issue, although the socio-economic context is partly implicated. Precarious living conditions expose individuals to an increased risk of ACEs, FI, and psychological distress [25]. However, food security issues appear to be a major clinical determinant, likely explaining a substantial proportion of the mental health difficulties observed in this population.

While mental health has been designated a major national cause in France for the second year [26], the needs of vulnerable young people and their specific challenges remain largely overlooked, as issues of social and professional integration are only beginning to be linked to psychological well-being [2]. The level of care provided by ML stays highly uneven across different local branches. However, a program recently piloted in several French territories demonstrated that providing early psychological support within integration pathways can help prevent suicide risk, particularly among young people without active psychiatric follow-up [27].

In 2022, the ML of Paris (MLP) expressed the desire to explore interrelationships between child maltreatment, FI and psychological distress [28], in light of the impacts observed among their youth.

It appears essential to better understand the profound relationships between all these variables and to place basic needs at the core of care, as food deprivation constitutes a serious lethal risk and significantly contributes to the deterioration of mental well-being [29]. These considerations gave rise to the MISAPSY project (Childhood maltreatment, food insecurity, psychological distress and professional integration among socioeconomically disadvantaged young adults). Conducted at the University of Lorraine by researchers from the INTERPSY laboratory (UR4432), in collaboration with University of Paris Cité, this research examines the complex relationships between ACE, FI, and psychological distress among vulnerable young people enrolled in a professional integration program at the ML. The ultimate objective is to develop replicable models of understanding that can inform the design of support programs. This study focuses on a specific and understudied population: young adults in precarious situations, many of whom have faced adverse experiences and social exclusion. By positioning access to food as a central clinical determinant, the study examines its associations with ACEs and current psychological distress. By addressing the interconnections between material deprivation and mental health vulnerability in this population, the study aims to fill a significant gap in both research and practice.

In this article, we present the methodological characteristics of this research, which is built upon a solid institutional partnership framework and grounded in the contextual realities of the field, tailored to the specific needs of the target population. The study adopts a multidimensional approach, structured around three complementary components: a quantitative cross-sectional study, a qualitative study, and an intervention-based comparative longitudinal study. These methodologies aim to achieve several objectives: (1) To identify factors associated with FI and their connections to various aspects of mental health and ACE. (2) To conduct a review of psychologists’ practices in MLs and explore their subjective representations of psychological support. (3) To understand the impact of the type of ACEs on the life trajectories and how it influences FI severity. (4) To compare the effects of two different support systems by observing the evolution of FI and psychological distress among participants.

## Method/design

This research project is designed around three distinct methodological approaches. These phases are complementary (see: Table 1).

- A cross-sectional study (study 1) based on a quantitative approach, consisting of the widespread distribution of standardized online questionnaires and scales (see: Measures) to young adults receiving support from any ML nationwide.
  The instruments facilitate a systematic collection of quantitative data and a multidimensional analytical framework to model the associations between psychological and social vulnerability factors. This design supports the identification of overarching statistical patterns.
- A qualitative study (study 2) intended for psychologists within MLs. This exploratory study offers a complementary perspective on understanding the experiences of youth at the MLs. By collecting data through verbatim analysis this study helps to identify some of the individual and institutional issues that influence the dynamics of care in these structures.
- An interventional longitudinal study (study 3) comparing two support programs. It is a multicenter, two-arm, parallel-group, single-blind randomized controlled trial. Participation is voluntary for young people experiencing severe FI and psychological distress, with a history of ACE, exclusively from MLP. The control group receives weekly food assistance for 12 months. The experimental group benefits from an intervention combining weekly food assistance for 12 months and twice-monthly psychological follow-up for 6 months. The study follows a superiority framework, hypothesizing that the combined intervention (food assistance plus psychological support) will lead to greater improvements in psychological distress and FI level compared to food assistance alone.

*Participants are assessed 4 times (T0: start of the program*,* T1: 3 months after the beginning*,* T2: end of the psychological intervention (6 months after the beginning)*,* T3: end of the program (12 months after the beginning). Each assessment period allows for the measurement of changes over time through standardized scales and semi-structured qualitative interviews. Detailed analyses will enable comparison of the specific effects of each intervention.*

**Table 1:** Summary table of the research methodology

	Study 1	Study 2	Study 3
Type of study Method	Quantitative Cross-sectional study using self-administered questionnaires	Qualitative Semi-structured interviews	Longitudinal Intervention programs Self-administered questionnaires and semi-structured interviews
Eligibility criteria	Participant aged 18–25 Currently being supported by the Mission Locale	Being a psychologist Currently working at the Mission Locale Have at least one young adult in their active caseload	Participant aged 18–25 Currently enrolled in an insertion program in the Mission Locale Answer “yes” at item 8 of the FIES PHQ-9 score > 15 GAD-7 score > 10 exposed to one or more ACEs (CTQ-SF)
Exclusion criteria	Minors Do not speak and read French Under Guardianship or Curatorship Unable to provide informed consent	Not having psychological follow-up at the Mission Locale Unable to provide informed consent	Minors Do not speak and read French Under Guardianship or Curatorship Unable to provide informed consent Currently receiving psychiatric care for an acute or chronic mental illness requiring regular specialized treatment Presenting a social or medical emergency situation incompatible with the study Having already participated in a similar study or being currently engaged in a structured psychological intervention protocol
Main Outcomes	Food Insecurity prevalence Mental health aspects (anxiety, depression, PTSD and CPTSD) Childhood trauma Psychological resources (defense mechanisms, resilience, perceived social support) Substances use Quality of sleep	Psychological practice and working conditions Organization of care Perspective on the young people encountered Sensitivity to issues of trauma and vulnerability	Childhood trauma Mental health aspects (anxiety, depression, PTSD and CPTSD) Food Insecurity level Defense mechanisms Anamnesis, current living conditions, adherence and perception of interventions
Analysis	Quantitative analysis	Thematic analysis	Quantitative and qualitative analysis

### Participants

#### Study 1

Cochran’s formula (Z² × p × (1-p) / E²) was applied to determine the required sample size. We based our calculations on a population size of 180,000 young people supported during the previous year (N-1). The margin of error was set at 5%, with a confidence level of 95%. The required sample size for this cross-sectional study is therefore 384 respondents.

#### Study 2

Our qualitative study will include a sample of 15 volunteer psychologists from MLs, which is a number suitable for the Consensual Qualitative Research (CQR) method. Nevertheless, inclusion will continue until data saturation is reached. We will consider data saturation to have been reached when the analysis of a new interview reveals no new themes.

#### Study 3

To our knowledge, our study is one of the first to explore this approach in a structured and longitudinal manner. Considering the lack of comparable studies among precarious young adults we have adopted a pragmatic approach instead of a power calculation. We aim to include a total of 70 volunteer participants, or 35 people in each group.

### Procedures

#### Study 1

- Recruitment: A nationwide promotion will be launched across all MLs in France through the National Union of Local Missions (Union Nationale des Missions Locales: UNML) and the Regional Association of Local Missions (Association Régionale des Missions Locales: ARML). A flyer is designed to be sent out or displayed within the MLs. Each ML involved will send emails to registered young people. It includes detailed information about the research, a link to access the study and inclusion criteria (see: Table 1). Upon accessing the study’s homepage, participants will find an introductory note presenting the study, its objectives, a contact point for further information, and a link to open the questionnaire. This will lead to a consent form. To the question, “Do you consent to participate in this research?” participants will be able to answer “Yes” or “No.” Participants who select “Yes” access the questionnaire. Those who select “No” will not be able to continue.

#### Study 2

- Recruitment: The qualitative study is addressed to psychologists currently working at the ML and having at least one young adult in their active caseload. A communication will be made by the UNML and ARML. After a presentation of the study by the researcher, the psychologists will receive an information sheet (see: supplementary material) detailing the study’s objectives, methodology, duration, constraints, and risks. The volunteer psychologists will then have 15 days to reflect and formalize their consent (see: supplementary material) by signing the dedicated form.
- Interview: After this period, each participant will be interviewed for a one-hour semi-structured session. The study can be conducted in person, via videoconference, or by telephone, according to the participant’s preference, but always under conditions that respect privacy. All interviews will be recorded using a voice recorder and will not collect any identifying data. The interviews will be transcribed, and the audio recordings destroyed after the topics have been validated by inter-rater agreement (according to the CQR method). Confidentiality will thus be guaranteed.

#### Study 3

**Fig. 1:**
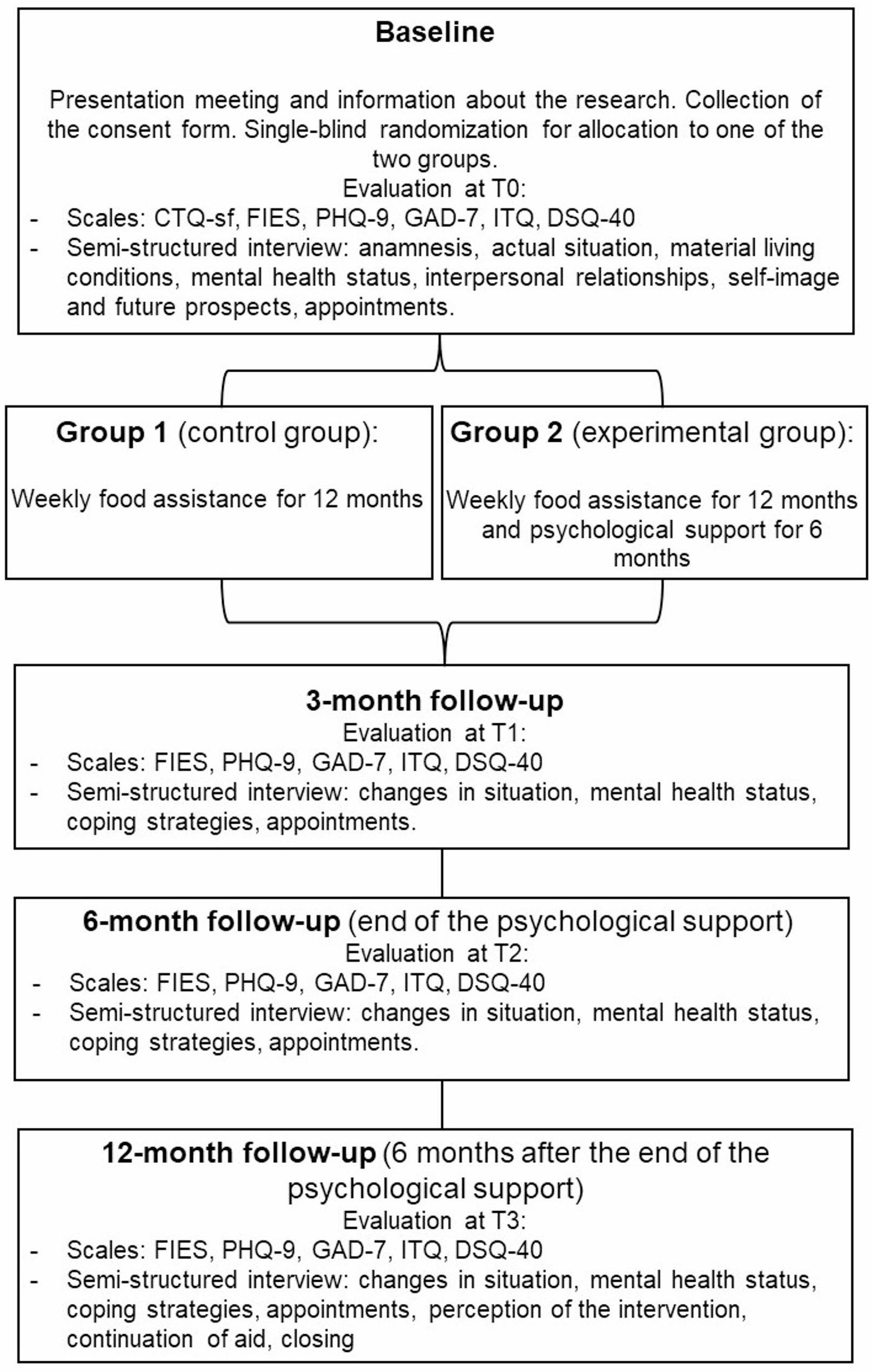
Procedure flowchart for longitudinal study (study 3). This diagram outlines participant enrolment, allocation to intervention arms, assessment time points, and follow-up over the 12-month study period

- Recruitment: The longitudinal study is intended for young people from MLP. Young people will be recruited through an explanatory email and the distribution of a flyer in the 6 MLP. Young people who have expressed interest will be interviewed by a member of the research team to verify their eligibility (see: Table 1), provide them with a detailed information sheet (see: supplementary material), and answer any questions. Eligible persons will then have 15 days to consider their decision and formalize their participation by signing the informed consent form (see: supplementary material).
- Randomization procedure: Participants will be assigned to one of two arms using a single-blind randomization procedure (1:1 allocation ratio). The assignment will be handled by a researcher who will not perform the data analysis, using a computer and a random number generator. The sequence will be stored in a secure file until participant enrollment is completed. Due to the nature of the intervention, neither the participants nor the facilitators (partner psychologists) can be blinded to group membership. However, the analysts of the outcome data will remain blinded to this group membership during statistical analyses. No formal unblinding procedure is planned, as there is no blinding at the participant level.
- Intervention and comparator: The control group will receive food assistance once a week for 12 months. The experimental group will receive the same food aid and in addition, psychological support twice a month for 6 months. The psychological support consists of individual sessions conducted by experienced psychodynamic psychologists and partners of the MLP. Food aid consists of distributing food baskets from MLP partner associations. The services are already offered by the MLP and are simply being reorganized within the framework of the study. In order not to deprive participants of potential care, the comparison of these two distinct approaches was guided by strong ethical considerations.
- Assessments and outcomes: These conditions enable the effects of different types of interventions to be measured within a supportive framework. Each participant will have 4 evaluation times during the year (T0, T1, T2, T3). Each time, they will first complete standardized scales and then have a semi-structured interview (see: Fig. 1). The interview will aim to characterize the participant’s experience, gather information about their history (T0), and assess their adherence to the proposed follow-up care, living conditions, interpersonal relationships, and future projections (T1, T2, T3). A closing session (T3) will also allow for a retrospective evaluation of the experience of the program, the maintenance of its effects, and the desire to continue the follow-up care. The primary outcome is the change in food insecurity level between baseline (T0) to 12 months (T3). Secondary outcomes include: (1) change in depressive symptoms (PHQ-9 score), change in anxiety symptoms (GAD-7 score), change in PTSD and CPTSD symptom dimensions (ITQ criteria) and change in defensive functioning (DSQ-40 scores). Adherence to food assistance and/or psychological support and any change in participant situation will be documented based on self-report during semi-structured interviews.

### Measures

#### Study 1

The questionnaire consists of a sociodemographic section follows by standardized scales. In order of presentation, the questionnaires are:

##### Childhood Trauma Questionnaire Short Form (CTQ-SF) [30, 31]

A 28-item instrument evaluating various types of childhood trauma, including emotional, physical and sexual abuse, and emotional and physical neglect. An additional item has been included in this scale assessing commercial child sexual exploitation.

##### Food Insecurity Experience Scale (FIES) [32–34]

An 8-item measure assessing the severity of food insecurity experienced, based on difficulties accessing adequate food.

##### Patient Health Questionnaire-9 (PHQ-9) [35, 36]

A 9-item tool used for depression screening that also provides an indication of the severity of depressive symptoms.

##### Generalized Anxiety Disorder (GAD-7) [37–39]

A 7-item self-report questionnaire designed to assess the patient’s anxiety levels over the previous two weeks.

##### International Trauma Questionnaire (ITQ) [40, 41]

An 18-item self-report measure of PTSD and CPTSD symptoms based on ICD-11 criteria. It includes clusters of PTSD, clusters of DSO and functional impairment for both clusters.

##### Insomnia Severity Index (ISI) [42, 43]

Sleep difficulties will be assessed using the first three items of the ISI (Item 1: a, b, c), which specifically address nighttime complaints: difficulty falling asleep, difficulty maintaining sleep, and early awakenings. Each item is rated on a five-point Likert scale ranging from 0 ("no problem") to 4 ("very severe problem"). The total score, obtained by summing the item scores, ranges from 0 to 12, with higher scores indicating more significant nighttime sleep difficulties.

To limit the administration workload and reduce conceptual overlap with other instruments in the protocol, only these three items were included. The dimensions relating to daytime impact, sleep-related distress and overall dissatisfaction, also assessed in the full version of the ISI, are in fact already partially taken into account by the Patient Health Questionnaire-9 (PHQ-9) and the Generalized Anxiety Disorder-7 (GAD-7), which respectively assess depressive symptoms, anxiety and impairments in functioning.

##### Alcohol Use Disorders Identification Test (AUDIT) [44, 45]

A 10-item questionnaire designed to identify individuals at risk of alcohol addiction, addressing alcohol consumption, dependence, and related problems.

##### Cannabis Abuse Screening Test (CAST) [46]

A 6-item screening tool to identify problematic cannabis use, particularly among adolescents and young adults, focusing on consumption habits and related issues over the past 12 months.

##### Fagerström Test for Nicotine Dependence [47, 48]

A 6-item questionnaire assessing the level of nicotine dependence in smokers.

##### Defense Style Questionnaire-40 (DSQ-40) [49, 50]

A 40-item questionnaire measuring 20 defensive mechanisms divided into three distinct styles: mature, neurotic or immature defenses.

##### Resilience Scale for Adults (RSA) [51]

A 33-item instrument evaluating protective resilience factors among adults.

##### Multidimensional Scale of Perceived Social Support (MSPSS) [52]

A 12-item questionnaire designed to measure perception of support in the dimensions of family, friends and significant other.

#### Study 2

A semi-structured interview guide is designed as an exploratory approach. This choice reflects the limited and fragmented body of research on psychological practices within the context of the ML. This is also in accordance with the diversity of possible practices in psychology and the existing inequalities in terms of working conditions through MLs. The study aimed to understand the subjective representations and situated practices of psychologists. An inductive and open-ended approach was deemed more appropriate. This design allows for the emergence of unexpected themes while avoiding the imposition of theoretical frameworks that might not fully account for the specific institutional and clinical realities of this context [53].

#### Study 3

##### Childhood Trauma Questionnaire Short Form (CTQ-SF) [30, 31]

A 28-item instrument evaluating various types of childhood trauma, including emotional, physical and sexual abuse, and emotional and physical neglect. An additional item has been included in this scale assessing commercial child sexual exploitation.

##### Food Insecurity Experience Scale (FIES) [32–34]

An 8-item measure assessing the severity of food insecurity experienced, based on difficulties accessing adequate food.

##### Patient Health Questionnaire-9 (PHQ-9) [35, 36]

A 9-item tool used for depression screening that also provides an indication of the severity of depressive symptoms.

##### Generalized Anxiety Disorder (GAD-7) [37–39]

A 7-item self-report questionnaire designed to assess the patient’s anxiety levels over the previous two weeks.

##### International Trauma Questionnaire (ITQ) [40, 41]

An 18-item self-report measure of PTSD and CPTSD symptoms based on ICD-11 criteria. It includes clusters of PTSD, clusters of DSO and functional impairment for both clusters.

##### Defense Style Questionnaire-40 (DSQ-40) [46, 47]

A 40-item questionnaire measuring 20 defensive mechanisms divided into three distinct styles: mature, neurotic or immature defenses.

### Data analysis

#### Study 1

The statistical analyses of the cross-sectional study will be performed using R software (RStudio), applying a significance level of 0.05. Descriptive and multivariate analyses (e.g., Chi-square/Fisher’s exact tests, correlation tests, ANOVA, etc.) will be used to characterize the population. The research team will also conduct correlation and factor analyses. In addition, we intend to perform a network analysis [54]. We will refer to the STROBE guidelines for epidemiological studies [55].

### Study 2

The analysis of the data from the semi-structured interviews will be conducted following a CQR approach. The themes will be identified by two researchers, using a structured and iterative procedure, aimed at the systematic search for a consensus among researchers. A third researcher, external to the data analysis, will be responsible for validating the categories in order to minimize primary group biases. The process will follow COREQ-32 recommendations [56, 57]. NVivo 15 software will be used.

### Study 3

The primary analysis will be based on repeated-effects linear mixed models, allowing assessment of the evolution of scores over time while taking into account intra-individual correlation and the longitudinal structure of the data. The group x time interaction, defined a priori, will be tested with a two-sided significance threshold set at α = 0.05, without correction for multiple comparisons. In addition, the research team will conduct: intergroup comparisons at several times (Student’s t-tests), intra-group comparison between two time points (paired t-test), exploratory analyses of subgroups based on sociodemographic or biographical variables. If data distribution is non-parametric, analyses such as Mann-Whitney, Wilcoxon, and Kruskal-Wallis tests will be employed. To limit the risk of type I errors in secondary and post-hoc analyses, a significance threshold adjustment will be applied in cases of multiple comparisons, using a Bonferroni correction (corrected α = 0.05 / m, where m corresponds to the number of tests performed within the same set of hypotheses). Exploratory analyses will be clearly identified as such and interpreted with caution, given the sample size.

Missing data will be taken into account based on their nature and frequency. If their proportion exceeds 5%, multiple imputation may be considered to limit bias, subject to the identified non-response mechanism [58].

All statistical analyses will be performed using the R software (RStudio).

### The roles of stakeholders of the research

The university researchers on the team are also experienced clinical psychologists working with young people (AE and MC). The University of Paris Cité is an institutional partner in the study. The partner organizations in the research project will contribute by disseminating the cross-sectional study online at the regional and national levels. The MLs in Paris are also contributing by offering services (food assistance, psychotherapist interventions) that we are using and adapting for the study.

The social work teams within each organization will facilitate the communication of information about the different phases of the study, invite the young people they support to participate, and host meetings with participants at their facilities. The scientific director of this research, AE, assumes overall responsibility for the project, including budget oversight and adherence to ethical procedures.

### Ethical considerations

We obtained approval from the Committee for the Protection of Persons (CPP) Ile de France XI to conduct this study, classified as a Minimal Risk Interventional Research (RIPH 2) study, and registered under number ID-RCB: 2025-A01928-41.

In addition, the MISAPSY project received a favorable opinion from the Ethics Committee (EC) of the Hôpitaux de Saint-Maurice (PR-2024-6) and the EC of the University of Lorraine (AE2025-0037). The research project is also registered in the data processing registry of the University of Lorraine under number 2025 − 462 and adheres to the Reference Methodology MR-001, French regulations defined by the National Commission for Information Technology and Civil Liberties (CNIL) govern research involving personal data in the health sector. In accordance with the General Data Protection Regulation (GDPR) and the University of Lorraine’s internal guidelines, these records ensure that the processing of participants’ personal data complies with ethical and legal data protection standards.

Any information that could identify participants will be removed before data analysis to guarantee their anonymity.

### Data monitoring and management

As data monitoring responsibilities are shared among the research team members, no data monitoring committee has been appointed. Data entry will be verified twice to ensure accuracy, and data quality will be assured through range checks. Participants will receive reminders. If they do not attend the appointments (study 3), reminders will be sent by text message or email. Data already collected from participants who have ended their participation will be retained for analysis. All data will be pseudonymized via a lookup table during the active phase of the study. After this, the lookup table will be destroyed to render the data completely anonymous. Considering the minimal-risk nature of the study, it will only be discontinued prematurely in case of unforeseen ethical concerns.

### Safety considerations

The instruments used in this study are standardized and validated questionnaires. They have been widely used in previous studies involving young and vulnerable populations, often through self-administration. Their use does not present any specific identified risks, apart from possible occasional discomfort when reflecting on certain personal situations.

Regarding the qualitative interviews in Study 2, no adverse effects are expected. However, the semi-structured interviews in Study 3 comprise a period for gathering information and subjective experiences, particularly during the initial meeting. Although conducted in a respectful and non-intrusive manner, these exchanges may potentially elicit emotions related to past or present events, especially in the context of a history of precarious living conditions, family instability, or childhood adversity.

To mitigate this risk, all interviews will be conducted by experienced psychologists trained in trauma clinical practice and in gathering information in vulnerable situations. In cases of obvious distress or a perceived need for more specific support, participants can be immediately referred to the psychologists at the ML.

If a psychiatric evaluation is deemed necessary a referral to a partner psychiatrist can be made. These resources are available regardless of the participant’s assigned group.

All of these measures help minimize potential psychological risks and ensure rapid and appropriate support when needed. If an exclusion criterion is met during the study, participation will be stopped.

Withdrawal of participants is entirely voluntary and will have no consequences, thus ensuring adherence to ethical research principles. Withdrawal of consent during data collection constitutes termination of participation. Participants who cease participating in research interviews or discontinue their participation in the support program will be considered lost to follow-up.

## Discussion

The objective of this study is to better understand the issues related to FI among precarious young adults and its associations with ACE, trauma and psychological distress. The aim is to adapt support systems in MLs services and to expand access to care. By focusing on a population facing multiple and cumulative forms of vulnerability, this research seeks to contribute to a more integrated and clinically relevant understanding of the links between material deprivation, trauma, and mental health. The results are expected to inform both scientific knowledge and professional practices by modeling the interrelationships between FI, ACEs, and psychological symptoms, while supporting the development of accessible and appropriate care pathways within these institutions.

We expect the results to highlight a significant prevalence of ACEs, trauma-related symptoms, and psychological distress among young people experiencing FI. We hypothesize that FI is not merely a contextual or socioeconomic correlate of psychological distress, but may constitute a central clinical determinant that interacts with earlier traumatic experiences. From this perspective, FI may reflect disruptions in the regulation of basic needs rooted in childhood adversity. This deregulation appears to be a determining factor in mental health.

Previous studies have demonstrated the impact of traumatic experiences on the development and persistence of FI, as well as their combined impact on psychological suffering. These findings underscore the importance of considering FI as a chronic stressor that exacerbates psychological suffering, rather than as a secondary or peripheral issue [59, 60]. Furthermore, FI is not always an individual problem. When a family is affected, the consequences impact both parents and children [61, 62]. In line with this literature, our study emphasizes the need to place basic needs at the heart of mental health care, as psychological interventions are likely to be limited in their effectiveness when conducted in contexts of ongoing material deprivation. Addressing FI may therefore represent a necessary, though not sufficient, condition for psychological stabilization and recovery [63, 64].

A central contribution of this research lies in its multidimensional and mixed-methods design. The planned network analyses may help clarify the role of FI within complex symptom systems [54]. This approach allows for a nuanced understanding of how material and psychological factors interact dynamically, in line with lived experience. By identifying central and bridging elements within symptom networks, this perspective may help clarify how material conditions and psychological processes interact and mutually influence one another over time [65].

In parallel, the qualitative and exploratory dimensions of the study are expected to provide insight into how FI and psychological suffering are perceived, interpreted, and managed in everyday professional practice within ML services. Given the heterogeneity of practices and institutional constraints across these structures, this exploratory approach is essential to understanding how young people’s suffering is perceived and addressed in everyday clinical work. These findings are expected to highlight gaps between identified needs and available responses, as well as tensions between social support, emergency aid, and psychological care, thereby informing future adaptations of support systems [66].

Finally, the longitudinal comparative intervention study aims to assess the respective and combined effects of food assistance and psychological support. We expect that while food aid alone may lead to short-term improvements in distress, the combination of food security and sustained psychological follow-up will result in greater and more durable reductions in psychological symptoms, particularly those related to trauma. This study may also shed light on differential trajectories according to the type of ACE, suggesting the need for more tailored, trauma-informed interventions [67]. We think that trauma-focused care could generate significant improvements, in addition to meeting unmet needs [68].

Ultimately, this research project aims to support the development of comprehensive, evidence-based prevention and intervention strategies that acknowledge the inseparability of material conditions and mental health. By positioning food insecurity as a central clinical and public health issue, MISAPSY seeks to promote more holistic, equitable, and transferable models of care for vulnerable young adults within MLs’ services.

## Supplementary Information

Supplementary Material 1.

## Data Availability

The datasets generated and/or analysed during the current study are not publicly available in accordance with GDPR-compliant procedures and the reference methodology MR-001 (Commission Nationale de l’Informatique et des Libertés) to ensure confidentiality. The data collected will be anonymized and stored on secure servers at the University of Lorraine with an access strictly limited to authorized members of the MISAPSY project research team. The study results will be published as open data and shared with the scientific community through various channels, including scientific publications, posters, and oral presentations at national and international conferences. Participants will be informed of the study results by their ML.

## Abbreviations

MISAPSY: Childhood maltreatment, food insecurity, psychological distress and professional integration among socioeconomically disadvantaged young adults
PTSD: Post-Traumatic Stress Disorder
CPTSD: Complex Post-Traumatic Stress Disorder
ML: Mission Locale
FI: Food Insecurity
ACE: Adverse Childhood Experience
MLP: Mission Locale de Paris
CQR: Consensual Qualitative Research
UNML: Union Nationale des Missions Locales
ARML: Association Régionale des Missions Locales
CTQ-SF: Childhood Trauma Questionnaire Short Form
FIES: Food Insecurity Experience Scale
PHQ-9: Patient Health Questionnaire-9-item scale
GAD-7: Generalized Anxiety Disorder 7-item scale
ITQ: International Trauma Questionnaire
DSO: Disorders in Self Organization
ISI: Insomnia Severity Index (3-item version)
AUDIT: Alcohol Use Disorders Identification Test
CAST: Cannabis Abuse Screening Test
DSQ-40: Defense Style Questionnaire-40-item scale
RSA: Resilience Scale for Adults
MSPSS: Multidimensional Scale of Perceived Social Support
ANOVA: Analysis of Variance
STROBE: Strengthening the Reporting of Observational Studies in Epidemiology
COREQ-32: Consolidated criteria for reporting qualitative research (32-item checklist for interviews and focus group)
CPP: Committee for the Protection of Persons
EC: Ethical Committee
CNIL: Commission for Information Technology and Civil Liberties
GDPR: General Data Protection Regulation
