# Supplementary material for: MISAPSY: childhood maltreatment, food insecurity, psychological distress and professional integration among socioeconomically disadvantaged young adults – a mixed-methods study protocol
Source: BMC Psychol. 2026 Mar 13;14:576. doi: 10.1186/s40359-026-04292-0 (PMC13097724; doi:10.1186/s40359-026-04292-0)
Supplement: Supplementary file 1 — Supplementary Material 1. [file 40359_2026_4292_MOESM1_ESM.docx]

**Supplementary Material**

**Table A:** Spirit guidance

**SPIRIT 2025 checklist of items to address in a randomized trial protocol***

| **Section / Topic** | **No** | **SPIRIT 2025 checklist item description** | **Reported on page no.** |
| --- | --- | --- | --- |
| **Administrative information** | | |  |
| Title and structured summary | 1a | Title stating the trial design, population, and interventions, with identification as a protocol | N°1 |
|  | 1b | Structured summary of trial design and methods, including items from the World Health Organization Trial Registration Data Set | N°2 |
| Protocol version | 2 | Version date and identifier | N°2/17 |
| Roles and responsibilities | 3a | Names, affiliations, and roles of protocol contributors | N°1 / 18 |
|  | 3b | Name and contact information for the trial sponsor | N°4 |
|  | 3c | Role of trial sponsor and funders in design, conduct, analysis, and reporting of trial; including any authority over these activities | N°18 |
|  | 3d | Composition, roles, and responsibilities of the coordinating site, steering committee, endpoint adjudication committee, data management team, and other individuals or groups overseeing the trial, if applicable | / |
| **Open science** | | |  |
| Trial registration | 4 | Name of trial registry, identifying number (with URL), and date of registration. If not yet registered, name of intended registry | N° 2 |
| Protocol and statistical analysis plan | 5 | Where the trial protocol and statistical analysis plan can be accessed | N°2 / 17-18 |
| Data sharing | 6 | Where and how the individual de-identified participant data (including data dictionary), statistical code, and any other materials will be accessible | N° 17-18 |
| Funding and conflicts of interest | 7a | Sources of funding and other support (e.g., supply of drugs) | N°18 |
|  | 7b | Financial and other conflicts of interest for principal investigators and steering committee members | N°18 |
| Dissemination policy | 8 | Plans to communicate trial results to participants, healthcare professionals, the public, and other relevant groups (e.g., reporting in trial registry, plain language summary, publication) | N°18 |
| **Introduction** | | |  |
| Background and rationale | 9a | Scientific background and rationale, including summary of relevant studies (published and unpublished) examining benefits and harms for each intervention | N°3 |
|  | 9b | Explanation for choice of comparator | N°4 |
| Objectives | 10 | Specific objectives related to benefits and harms | N°5 |
| **Methods: Patient and public involvement, trial design** | | |  |
| Patient and public involvement | 11 | Details of, or plans for, patient or public involvement in the design, conduct, and reporting of the trial | 13 |
| Trial design | 12 | Description of trial design including type of trial (e.g., parallel group, crossover), allocation ratio, and framework (e.g., superiority, equivalence, non-inferiority, exploratory) | N°5 / Table 1 |
| **Methods: Participants, interventions, and outcomes** | | |  |
| Trial setting | 13 | Settings (e.g., community, hospital) and locations (e.g., countries, sites) where the trial will be conducted | N°5 |
| Eligibility criteria | 14a | Eligibility criteria for participants | Table 1 |
|  | 14b | If applicable, eligibility criteria for sites and for individuals who will deliver the interventions (e.g., surgeons, physiotherapists) | / |
| Intervention and comparator | 15a | Intervention and comparator with sufficient details to allow replication including how, when, and by whom they will be administered. If relevant, where additional materials describing the intervention and comparator (e.g., intervention manual) can be accessed | N°7 |
|  | 15b | Criteria for discontinuing or modifying allocated intervention/comparator for a trial participant (e.g., drug dose change in response to harms, participant request, or improving/worsening disease) | N°14-15 |
|  | 15c | Strategies to improve adherence to intervention/comparator protocols, if applicable, and any procedures for monitoring adherence (e.g., drug tablet return, sessions attended) | N°9 |
|  | 15d | Concomitant care that is permitted or prohibited during the trial | N°14-15 ; Table 1 |
| Outcomes | 16 | Primary and secondary outcomes, including the specific measurement variable (e.g., systolic blood pressure), analysis metric (e.g., change from baseline, final value, time to event), method of aggregation (e.g., median, proportion), and time point for each outcome | N°7-13 |
| Harms | 17 | How harms are defined and will be assessed (e.g., systematically, non-systematically) | N°14-15 |
| Participant timeline | 18 | Time schedule of enrollment, interventions (including any run-ins and washouts), assessments, and visits for participants. A schematic diagram is highly recommended (see Figure) | N°7; fig. 1 |
| Sample size | 19 | How sample size was determined, including all assumptions supporting the sample size calculation | N°6-7 |
| Recruitment | 20 | Strategies for achieving adequate participant enrollment to reach target sample size | N°7 |
| **Methods: Assignment of interventions** | | |  |
| Randomization: |  |  |  |
| Sequence generation | 21a | Who will generate the random allocation sequence and the method used | N°8-9 |
|  | 21b | Type of randomization (simple or restricted) and details of any factors for stratification. To reduce predictability of a random sequence, other details of any planned restriction (e.g., blocking) should be provided in a separate document that is unavailable to those who enroll participants or assign interventions | N°8 |
| Allocation concealment  mechanism | 22 | Mechanism used to implement the random allocation sequence (e.g., central computer/telephone; sequentially numbered, opaque, sealed containers), describing any steps to conceal the sequence until interventions are assigned | N°8 |
| Implementation | 23 | Whether the personnel who will enroll and those who will assign participants to the interventions will have access to the random allocation sequence | N°8 |
| Blinding | 24a | Who will be blinded after assignment to interventions (e.g., participants, care providers, outcome assessors, data analysts) | N°8 |
|  | 24b | If blinded, how blinding will be achieved and description of the similarity of interventions | N°8 |
|  | 24c | If blinded, circumstances under which unblinding is permissible, and procedure for revealing a participant’s allocated intervention during the trial | / |
| **Methods: Data collection, management, and analysis** | | |  |
| Data collection methods | 25a | Plans for assessment and collection of trial data, including any related processes to promote data quality (e.g., duplicate measurements, training of assessors) and a description of trial instruments (e.g., questionnaires, laboratory tests) along with their reliability and validity, if known. Reference to where data collection forms can be accessed, if not in the protocol | N°7/10/12 /14 |
|  | 25b | Plans to promote participant retention and complete follow-up, including list of any outcome data to be collected for participants who discontinue or deviate from intervention protocols | N°14 |
| Data management | 26 | Plans for data entry, coding, security, and storage, including any related processes to promote data quality (e.g., double data entry; range checks for data values). Reference to where details of data management procedures can be accessed, if not in the protocol | N°14 |
| Statistical methods | 27a | Statistical methods used to compare groups for primary and secondary outcomes, including harms | N°12 |
|  | 27b | Definition of who will be included in each analysis (e.g., all randomized participants), and in which group | N°12 |
|  | 27c | How missing data will be handled in the analysis | N°12-13 |
|  | 27d | Methods for any additional analyses (e.g., subgroup and sensitivity analyses) | N°12-13 |
| **Methods: Monitoring** | | |  |
| Data monitoring committee | 28a | Composition of data monitoring committee (DMC); summary of its role and reporting structure; statement of whether it is independent from the sponsor and funder; conflicts of interest and reference to where further details about its charter can be found, if not in the protocol. Alternatively, an explanation of why a DMC is not needed | N°14 |
|  | 28b | Explanation of any interim analyses and stopping guidelines, including who will have access to these interim results and make the final decision to terminate the trial | N°14 |
| Trial monitoring | 29 | Frequency and procedures for monitoring trial conduct. If there is no monitoring, give explanation | N°14 |
| **Ethics** | | |  |
| Research ethics approval | 30 | Plans for seeking research ethics committee/institutional review board approval | N°14/17 |
| Protocol amendments | 31 | Plans for communicating important protocol modifications to relevant parties | N°17 |
| Consent or assent | 32a | Who will obtain informed consent or assent from potential trial participants or authorized proxies, and how | N°7/17 |
|  | 32b | Additional consent provisions for collection and use of participant data and biological specimens in ancillary studies, if applicable | / |
| Confidentiality | 33 | How personal information about potential and enrolled participants will be collected, shared, and maintained in order to protect confidentiality before, during, and after the trial | N°14 |
| Ancillary and post-trial care | 34 | Provisions, if any, for ancillary and post-trial care, and for compensation to those who suffer harm from trial participation | / |

*We strongly recommend reading this checklist in conjunction with the SPIRIT 2025 Explanation and Elaboration and the SPIRIT 2025 Expanded Checklist for important clarifications on all the items. We also recommend reading relevant SPIRIT extensions. See [www.consort-spirit.org](http://www.consort-spirit.org)

Citation: Chan A-W, Boutron I, Hopewell S, Moher D, Schulz KF, et al. SPIRIT 2025 statement: updated guideline for protocols of randomised trials. BMJ 2025;389:e081477. <https://dx.doi.org/10.1136/bmj-2024-081477>

© 2025 Chan A-W et al. This is an Open Access article distributed under the terms of the Creative Commons Attribution License (<https://creativecommons.org/licenses/by/4.0/>), which permits unrestricted use, distribution, and reproduction in any medium, provided the original work is properly cited.

Appendix 1

**Information notice (qualitative study)**

**Project MISAPSY**

**N° ID-RCB : 2024-A02662-45**

Read this information notice carefully and ask any questions you may have. You can then be able to decide whether or not you want to participate in this research.

**Research project:** “Childhood Maltreatment, Food Insecurity, Psychological Distress and Professional Integration among Socioeconomically Disadvantaged young adults.”

This psychology research project, is led by Aziz Essadek, a member of the INTERPSY laboratory (http://interpsy.univ-lorraine.fr), who is the scientific director. It is being conducted in partnership with the University of Lorraine and the network of Missions Locales de Paris, and is funded by the Hospital Foundation for Research. This research has received approval from the Ethics Committee.

This six-month research project aims to understand your professional perspective on supporting young people at the Mission Locale (Local Employment Center), and more specifically, the challenges related to addressing certain mental health conditions.

We have developed a semi-structured interview guide to explore various aspects of your professional experience with these vulnerable young people. An exploratory thematic analysis will be based on the data collected.

**Your decision to participate, refuse to participate, or withdraw from the study will have no impact on your relationships with any parties involved in this research, nor on your regular work.**

**Procedure if you choose to participate:** Your participation is voluntary and based on your consent; therefore, you will receive an informed consent form. You will then have two weeks to decide whether or not to consent to the study by signing the form.

The study consists of a single interview of approximately one hour with a member of the research team. Several themes will be addressed, based on a pre-prepared interview guide, in a semi-structured manner. You will be able to speak freely on these different topics related to the young people of the Mission Locale and to your work at the Mission Locale.

The interviews will be recorded using a dedicated dictaphone for the study, so that they can be transcribed verbatim. For the purposes of this research, your personal data will be processed in accordance with the provisions of the General Data Protection Regulation (GDPR) No. 2016/679 and the amended French Data Protection Act (Law No. 78-17). All data concerning you will remain strictly confidential. They will be pseudonymized so that no information directly identifying you is available. Consent forms and identifying data will be destroyed at the end of the study.

Audio recordings will be destroyed after transcription and inter-rater validation (no later than June 2027).

Remaining data, such as anonymized transcripts of audio recordings, will be kept for up to two years after the final publication of results.

You may withdraw from the study at any time, without justification or consequence, and request that your previously collected data be destroyed.

**Benefits and constraints:** We expect this study to have overall benefits, including scientific insights that will allow for adjustments to the support offered by the Mission Locale and potentially even a public health impact.

For users of the Mission Locale, the primary benefit of this study will be improved support that addresses specific and complex issues such as psychological trauma.

For professionals, the benefits could include adjustments to their practices in line with the topics covered. Improved support from the organization for managing complex, multifactorial situations could also be a positive outcome.

Regarding risks, the interviews may bring up difficult experiences encountered while supporting young people at the Mission Locale. If your participation cause distress or disruption, you can count on the support and understanding of the investigator/psychologist conducting the interview, who will be able to assist you. Support services may also be offered.

This research will result in scientific publications available online via Open Access. Feedback on the overall results can be provided by the Mission Locale or, if you prefer, by email.

For further information, please contact: Aziz Essadek, Scientific Director and Coordinating Investigator (aziz.essadek@univ-lorraine.fr) or Maud Cappelletti, PhD candidate and Investigator (maud.cappelletti@univ-lorraine.fr).

Thank you very much for your potential participation in this study. We wish you all the best.

In accordance with the provisions of the General Data Protection Regulation No. 2016/679 (GDPR) and Law No. 78-17 known as "Data Processing and Freedoms", you have the right to access, rectify, object to, erase, limit and port your data. To exercise these rights, you can contact Maud Cappelletti at maud.cappelletti@univ-lorraine.fr or Aziz Essadek at aziz.essadek@univ-lorraine.fr.

The University of Lorraine has appointed a Data Protection Officer whom you can contact for any information at the following address: dpo-contact@univ-lorraine.fr (Legal Affairs Department, 34 Cours Léopold, 54052 Nancy Cedex, France).

For any questions relating to data protection or complaints concerning the processing of your personal data: dpo-contact@univ-lorraine.fr (Legal Affairs Department – ​​University of Lorraine, 34 Cours Léopold, BP 25233 - 54052 Nancy, France) or contact the supervisory authority in France, namely the Commission Nationale de l’Informatique et des Libertés (CNIL), at this address: https://www.cnil.fr/fr/contacter-la-cnil-standard-et-permanences-telephoniques or CNIL, 3 Place de Fontenoy, Complaints Department, TSA 80715, 75334 PARIS CEDEX 7)

Appendix 2

**Information notice (interventional study)**

**PRESPA Project / ID-RCB No.: 2025-A01928-41**

Please read this notice carefully and ask any questions you may have now or at any time.

Hello, here is some information regarding the research:

“Psychosocial intervention with young people in precarious situations: a longitudinal comparative study on psychological distress and food insecurity.”

This is a psychology research project, led by Ariane Bazan, who is the scientific director. It is being conducted in partnership with the University of Lorraine and the network of Missions Locales de Paris and is funded by the Hospital Foundation for Research.

Young people from the Mission Locale sometimes have difficulty accessing sufficient and/or healthy food. This can impact their psychological well-being. We believe that having experienced childhood adversity exacerbates this phenomenon. You are being contacted because your situation corresponds to this type of difficulty. The main objective of this study is to compare two different support programs by observing the evolution of the mental health of participants in each group, using questionnaire scores and thematic discourse analysis.

We also aim to better understand the effects of child maltreatment on food insecurity among young people receiving services from the Mission Locale, in relation to psychological distress. To this end, we are using both questionnaires and interviews.

We wish to recruit at least 70 young people. You cannot participate in the study if: you refuse to participate or are unable to provide informed consent due to impaired comprehension or communication skills; you are currently receiving psychiatric care for an acute or chronic mental illness requiring regular specialized treatment; you are experiencing a social or medical emergency that is incompatible with the study (e.g., unstable emergency accommodation, acute suicidal crisis, ongoing hospitalization); you are incarcerated, under guardianship, or under curatorship without specific legal authorization to participate in research; you have already participated in a similar study or are currently engaged in a structured psychological intervention protocol; or you have insufficient command of the French language, preventing you from understanding instructions, questionnaires, or interviews. The research is exploratory (exploring a little-studied phenomenon), multicenter (conducted simultaneously at different sites), and longitudinal: participants are followed for 12 months with four assessment points to observe their progress. It consists of comparing two different types of support. Participants are therefore randomly assigned to one of the following two groups (this is the randomization process): a 50% chance of being in group 1 and a 50% chance of being in group 2.

| For 6 months | |
| --- | --- |
| **Group 1**   - Food assistance: 1x/week (24 times) | **Group 2**   - Food assistance: 1x/week (24 times) - Appointment with a psychologist: 2x/months (12 sessions) |

In practical terms, whether you are in group 1 or 2, you will receive food assistance every week for the entire duration of the study, that is, the 6 months described above and the 6 months following (12 months in total). This assistance consists of a food basket sourced from a solidarity-based, anti-waste redistribution network. It will be delivered at your local Mission Locale by the associations Linkee or HopHopFood.

If you are in group 2, in addition to food assistance, you will have 6 months of psychological support appointments with a psychologist from the association parADOxes, at your Mission Locale. The psychological support will be tailored by the psychologist to each participant's specific needs in order to promote well-being. This may include reducing symptoms such as depression or anxiety, or providing support towards food security, among other things.

In addition, throughout your program, you will meet with a member of the research team 3 times and a 4th time 6 months later:

During 6 months of food aid

Or 6 months after the end of psychological support

During 6 months of follow-up

4^th^ appt

3^rd^ appt

2^nd^ appt

1^st^ appt

Each interview lasts approximately one hour. The first part of the interview consists of completing several mental health questionnaires (for example, a depression scale, an anxiety scale with multiple questions and answer choices). There will also be a scale on food insecurity and another on your reactions to life events. During the first interview, a questionnaire will assess the type and frequency of childhood maltreatment experienced. Between the first and third interviews, there will also be a questionnaire on the symptoms of simple and complex post-traumatic stress disorder. The second part of the interview is a discussion on topics predetermined by the investigator, concerning the participants' experiences.

For you, the benefits of this study will primarily be a reduction in food insecurity through food assistance and an overall improvement in well-being for both groups. This program will also allow you to experience the support services available. A significant reduction in psychological distress is the goal for Group 2. You will be able to continue these services after the study is completed.

The risks include the possibility that the interviews may touch upon difficult life periods and events. If your participation causes you distress, you can count on the support and compassion of the investigator/psychologist conducting the interview, who will be able to assist you. You can also contact your Mission Locale advisor.

Therefore, we ask that you commit to attending all scheduled appointments. This requires commitment and time and may be demanding.

- If you choose to withdraw your participation, no new data will be collected, but the data already collected may be retained and processed within the framework of this study.

- If we do not hear from you despite our follow-up attempts, we will consider your participation withdrawn.

- The investigator may terminate your participation at any time if conditions prevent the proper conduct of the study (for example, experiencing a social or medical emergency incompatible with the study, or being incarcerated, etc.). In this case, you will be notified of this decision, and the data already collected will be retained and processed within the framework of this study.

The data we collect is:

- Paper questionnaires that we digitize on secure servers and store under lock and key in a research office at the University of Lorraine.

- Audio recordings of the interviews, collected on a dictaphone dedicated to the study. These will be transcribed verbatim, and the audio recordings will be destroyed no later than the end of the data collection phase (12 months). When not in use, the dictaphone is stored under lock and key in the University of Lorraine's research office. The transcriptions are stored on a secure server at the University of Lorraine.

- Your data will remain strictly confidential. Your information will be pseudonymized using a lookup table with random number assignment for the 12 months of the study, ensuring that no information can directly identify you and that only the investigator will be able to retrieve your details.

- The consent forms will be destroyed at the end of the study (12 months plus the data analysis period). The lookup table will also be destroyed at the end of the study to ensure that the data is completely anonymized.

Thank you very much for your potential participation in this study. We wish you all the best.

Other legal aspects:

This research has been approved by the Ethics Committee (Committee for the Protection of Persons).

You have two weeks to decide whether or not to participate by signing the attached consent form. Your decision to participate, refuse to participate, or withdraw from the study will have no impact on you or your care at the Mission Locale. You may withdraw from the study at any time, without providing a reason.

We expect this study to allow the Mission Locale to adapt the support provided to the young people it assists. This could potentially be extended to other services that support similar populations and lead to a reorganization of the entire healthcare system. This research will result in scientific publications available online. Feedback on the overall results can be sent to you by the Mission Locale or, if you prefer, by email.

Insurance from HDI Global SE (via BiomedicInsure) covers the study.

All participants must be registered with the French social security system.

There is no waiting period before participants can take part in another study.

For more information, please contact: Aziz Essadek, Principal Investigator (aziz.essadek@u-paris.fr) or Maud Cappelletti, PhD student and PhD investigator (maud.cappelletti@univ-lorraine.fr).

In accordance with the GDPR, your data is processed on the legal basis of the performance of a task carried out in the public interest and in accordance with the necessity of processing health data for scientific purposes. Your personal identifying data, necessary for the study, is transmitted in coded form (pseudonymization) to the sponsor for computer processing to analyze the research results in relation to the objective presented to you. In this form, he can transmit them, in Europe, to organizations collaborating in research as well as to French or foreign Health Authorities.

In accordance with the provisions of the French Data Protection Act (Loi Informatique et Libertés), specifically CNIL regulation MR001: You have the right to access and rectify your personal data. You can exercise this right by contacting the investigator who is supervising you in the research and who is the only one who knows your identity.

It is possible that you may not be able to exercise certain rights provided for by the GDPR (restriction, erasure) if doing so would compromise the achievement of the study's objectives.

You have the right to object to the transmission of data covered by professional secrecy that may be used and processed within the framework of this research.

If you decide to withdraw from the study, the coded data collected before your withdrawal will be processed along with the other data collected for the study, but no new data concerning you will be collected or processed. In this case, your data will not be used at any time or for any other research.

The data will be kept for up to two years after the last publication related to this study (anonymized data) and archived at the Interpsy Laboratory (UR 4432) of the University of Lorraine.

The research sponsor is the University of Lorraine, 34 Cours Léopold, 54052 Nancy, France.

If you encounter any difficulties exercising your rights, you can contact the sponsor's Data Protection Officer (DPO) at the following email and postal address: dpo-contact@univ-lorraine.fr (Legal Affairs Department – ​​University of Lorraine, 34 Cours Léopold, BP 25233 - 54052 Nancy, France).

You have the right to lodge a complaint with the supervisory authority, the CNIL (French Data Protection Authority), at www.cnil.fr/plaintes or CNIL, 3 Place de Fontenoy, TSA 80715, 75334 Paris Cedex 07, France.

Appendix 3

**Consent Form**

**PRESPA Project**

**ID-RCB No.: 2025-A01928-41**

Please read this form carefully and ask any questions you may have. You will then be able to decide whether or not you wish to participate in this study/research.

**Study Title:** Psychosocial Intervention with Vulnerable Youth: A Longitudinal Comparative Study on Psychological Distress and Food Insecurity - PRESPA

Sponsor Name and Address: **University of Lorraine**, 34 Cours Léopold, 54000 Nancy, France

I,……………………………………………………………………………………………………………………………………………… (first and last name in capital letters), declare that I have understood the purpose and procedures of this study, which have been fully explained to me by ………...………….………………………………………

□ I have received the information sheet, which I have had the opportunity to review and which specifies the contact information for the investigator to be contacted with any questions.

□ All my questions were answered.

□ I was given two weeks to consider my decision.

□ I agree to participate in this research under the conditions specified in the attached information sheet. I am free to withdraw from the study at any time without this affecting my relationships with the study stakeholders. I will then inform the investigator or the lead scientist.

□ I have been informed that, in accordance with regulations on clinical trials, the Committee of the Persons’ Protection has issued a favorable opinion for the conduct of this study.

□ I understand that my data will be processed and protected in accordance with the European General Data Protection Regulation (GDPR) and the French Data Protection Act (CNIL). I agree that the data recorded during this research may be processed automatically by the sponsor or on its behalf, as described in the information letter, and that my rights concerning my coded personal data can be exercised at any time by contacting the investigator who is monitoring my progress in the research and who knows my identity, or the sponsor's Data Protection Officer (DPO).

□ I have been fully informed of the purpose of the processing (I have been informed what this data will be used for) as well as the recipients of this data.

□ I give my consent to participate in this research and to the processing of my personal data.

□ I may request any further information at any time from the study organizers, using the contact details provided in the information leaflet/information letter.

□ Since the study requires an audio recording:

□ I authorize such recording to be carried out according to the procedures specified in the information leaflet/letter.

□ I understand that the audio recording will only be kept for the time necessary to transcribe it.

□ I understand that the retention period for the transcript, in anonymized form, is limited to two years after the end of the

study.

□ I freely consent to participate in this study under the conditions explained to me.

My consent does not in any way release the investigator and the sponsor from all their responsibilities, and I retain all my rights guaranteed by law.

At the end of the research, I will be informed of the overall results of this research.

| TO BE COMPLETED BY THE PARTICIPANT |
| --- |
| Date: ………………  Participant’s signature: |

| TO BE COMPLETED BY THE INVESTIGATOR | |
| --- | --- |
| I,…………………………………………………………………………………………. (first and last name in capital letters), confirm that I have fully explained the objectives, methods and the potential risks to the participant. I commit to respecting the terms of this consent form, balancing respect for individual rights and freedoms with the requirements of scientific work.  Investigator’s telephone number: …………………………………….. | |
| Investigator’s signature: | Date: …………………………….. |

Made in duplicate, one copy to be kept by the investigator and the other given to the participant.
